# GCP-YOLO: high-precision detection of tiny chili flowers in complex greenhouse scenes

**DOI:** 10.3389/fpls.2026.1879612

**Published:** 2026-07-06

**Authors:** Minqiu Kuang, Yushi Wang, Xiaojian Li, Xiangjun Zou, Dawei Liu, Yang Xiang, Sebastian Bader, Fangping Xie, Yuxuan Zhang, Xu Li

**Affiliations:** 1 College of Electrical and Mechanical Engineering, Hunan Agricultural University, Changsha, China; 2 Foshan-Zhongke Innovation Research Institute of Intelligent Agriculture and Robotics, Foshan, China; 3 Hunan Provincial Key Laboratory of Intelligent Agricultural Machinery Equipment, Changsha, China; 4 Department of Computer and Electrical Engineering, Mid Sweden University, Sundsvall, Sweden; 5 College of Intelligent Science and Engineering, Beijing University of Agriculture, Beijing, China

**Keywords:** automated monitoring, chili flower detection, deep learning, plant phenotyping, protected horticulture, YOLO

## Abstract

Precise identification of chili flowers and buds is essential for enabling intelligent robotic pollination, continuous crop monitoring, and early yield prediction in protected horticulture. Reliable visual sensing remains challenging because chili targets are extremely small, densely distributed, and frequently occluded by foliage, while greenhouse environments introduce strong illumination variations and background reflections. These factors often lead to insufficient feature extraction and unstable detection accuracy in existing models, limiting their practical deployment in automated monitoring systems. To address these challenges, this study proposes GCP-YOLO, a lightweight yet high-performance detection framework built upon the YOLOv11n architecture. The model enhances small-target perception through three key improvements. First, a redesigned Generalized Feature Pyramid Network (GFPN) strengthens cross-scale feature interaction, improving the fusion of fine-grained texture cues and deep semantic information. Second, a C2CGA context-guided attention module is introduced to emphasize floral structural features while suppressing background noise caused by reflections and canopy clutter. Third, extended multi-scale detection heads (P2–P6) incorporate broader contextual information to reduce missed detections and false positives in dense planting scenarios. Experimental results on a custom chili flower dataset show that the proposed method achieves 92.8% precision, 83.7% recall, 90.8% mAP50, and 72.7% mAP50-95, improving upon the YOLOv11n baseline by 2.1, 1.3, 3.9, and 6.6 percentage points, respectively. Deployment on an NVIDIA Jetson AGX Orin edge platform demonstrates real-time inference at 97.9 FPS, confirming its suitability for on-device phenotyping. Overall, the proposed approach significantly improves detection robustness under complex greenhouse conditions, providing an effective visual sensing methodology for automated crop monitoring and data-driven yield estimation.

## 1 Introduction

As one of the most economically important vegetable crops worldwide, chili (*Capsicum annuum*) plays a crucial role in ensuring stable vegetable supply and supporting high-value protected agriculture production systems Kondo et al. (2025); Ollerton et al. (2014); Kuang et al. (2026c). In modern plant science and precision horticulture, the flowering stage is not only a key physiological phase determining fruit set and yield quality, but also an early indicator of production potential. Accurate monitoring of flowers and buds enables yield prediction several weeks ahead of harvest, providing valuable decision support for irrigation scheduling, pollination management, and labor planning Aquino et al. (2018); Ma et al. (2026); Kuang et al. (2026f).

Within high-throughput plant phenotyping pipelines, visual sensing has become a core component linking crop perception, autonomous robotics, and data-driven decision support systems Li et al. (2025a); Zhang et al. (2026b); Badar et al. (2025); Lu et al. (2026); Du et al. (2024); Bakhshayeshi et al. (2024); Cheng et al. (2024); Kuang et al. (2026a); Li et al. (2026). Compared with manual observation, automated vision-based monitoring can provide continuous, large-scale, and objective crop status information. Consequently, deep learning–based object detection methods, particularly YOLO-style and Transformer-based models, have rapidly become dominant solutions for agricultural visual sensing tasks due to their end-to-end inference capability and suitability for on-device and edge deployment Zhang et al. (2023); Li et al. (2024); Zhang et al. (2025); Li et al. (2021); Zhang et al. (2026c); Kuang et al. (2026d); Zhang et al. (2026a); Li et al. (2025b).

For crops with large and visually distinctive flowers, current detection systems have achieved considerable success, mainly focusing on balancing accuracy and computational efficiency. In apple orchards, Wu et al. Wu et al. (2020) reduced model complexity through channel pruning while maintaining detection accuracy. Shang et al. Shang et al. (2023) proposed a lightweight YOLOv5 variant based on ShuffleNetV2 and Ghost modules for growth-stage recognition. Sun et al. Sun et al. (2021) and Dias et al. Dias et al. (2018) further improved recognition accuracy by integrating semantic segmentation and shape constraints, while clustering-based methods Geng et al. (2025) and semi-supervised frameworks Wang et al. (2025) have been introduced to improve counting reliability with limited annotations. For coffee flowers, Sivasubramanian et al. Sivasubramanian et al. (2026) proposed an IoU-AI methodology based on Cascade R-CNN, which detects floral organs (stigma, anthers) and calculates their overlap to estimate pollen transmission, achieving 85.34%–94.77% detection accuracy across different blooming stages. Beyond apples, UAV-assisted detection systems have been developed for lotus flowers Lyu et al. (2024), while mobile sensing platforms and cascade CNNs have been applied to grape inflorescence counting and greenhouse tomato monitoring Palacios et al. (2020); Xu et al. (2022). Multi-hypothesis tracking approaches have also enabled temporal flower counting under partial occlusion Houtman et al. (2021). Recent studies have further promoted flower detection in complex agricultural environments. Deng et al. Deng et al. (2026) introduced a litchi flower detection framework integrating multi-scale attention and decoupled detection to enhance small-target recognition. Vilcapoma et al. Vilcapoma et al. (2025) employed convolutional neural networks for UAVbased marigold flower detection, demonstrating the effectiveness of aerial flower monitoring. Estrada et al. Estrada et al. (2024) developed a deep learning-based approach for flower detection and counting in densely populated peach orchards, achieving robust performance under crowded conditions.

However, these approaches often assume relatively distinguishable targets or moderate occlusion. In densely planted crops with complex canopy structures, such as kiwifruit or litchi orchards, severe overlap and small-scale targets significantly degrade detection reliability. To address this issue, recent studies have emphasized multi-scale feature fusion and attention mechanisms as key improvements. Guo et al. Li et al. (2022b, a) improved YOLO-based detectors for kiwifruit flowers, while Li et al. (2025c) proposed KiwiRecNet combining GAN-based enhancement with Transformer modules to capture fine-grained features under poor lighting. Li et al. (2023) further incorporated pose prediction for robotic pollination guidance. For other dense small targets, new loss functions and attention-enhanced YOLO variants Zhao et al. (2023) have been introduced to mitigate bounding box interference and background noise. Transformer-based detectors such as ELSF-DETR Xu et al. (2026) have also demonstrated the feasibility of capturing extremely small floral features with lightweight architectures.

Despite these advances, chili flower detection remains particularly challenging in greenhouse phenotyping scenarios. Compared with orchard flowers, chili buds are extremely small, densely distributed, and frequently occluded by foliage. Additionally, greenhouse conditions introduce strong specular reflections from leaves, plastic mulch, and structural materials, which often resemble floral textures. Existing studies have attempted to address these issues. Wang and Zhang (2025) incorporated CBAM attention, conditional information encoding, and MPDIoU loss into YOLOv8 to improve chili flower detection accuracy. Kuang et al. (2025b, 2026b, 2025a) proposed lightweight YOLO-based models with transfer learning, achieving high mAP while maintaining inference speed. Nevertheless, in highly cluttered greenhouse scenes, current models still struggle to simultaneously detect tiny buds and suppress visually similar background structures, resulting in frequent false positives and missed detections. These limitations hinder reliable large-scale deployment in automated greenhouse monitoring systems, where robustness, real-time inference, and compatibility with robotic platforms are essential.

To address these challenges, this study proposes GCP-YOLO, an optimized detection framework tailored for chili flower monitoring in greenhouse environments. Built upon the lightweight YOLOv11n backbone, the proposed model enhances small-target perception while preserving real-time performance for field and edge deployment. The main contributions are summarized as follows:

- Topology-aware feature fusion for tiny agricultural targets: A redesigned Generalized Feature Pyramid Network (GFPN) introduces cross-scale skip connections and weighted fusion strategies to strengthen the interaction between shallow texture features and deep semantic information, improving the detection of centimeter-scale buds in dense canopy scenes.
- Morphology-driven attention for greenhouse noise suppression: A novel C2CGA attention module integrates spatial and channel guidance to emphasize floral structural edges while suppressing reflections and background clutter typical of protected cultivation environments.
- Multi-scale contextual perception for robust crop monitoring: By extending the detection heads to P2–P6 levels, the model captures both local fine-grained features and global contextual information, enabling reliable detection in dense planting conditions and supporting downstream applications such as robotic pollination and early yield estimation.

Overall, this work provides a high-throughput visual sensing method for automated plant phenotyping in protected cultivation, offering a robust vision-based perception module for crop monitoring, decision support, and precision horticultural management.

## 2 Materials and methods

### 2.1 Image acquisition and dataset preprocessing

The data acquisition was conducted at the Vegetable Research Base of Hunan Agricultural University, located in Furong District, Changsha, Hunan Province, China (28°11’N, 113°04’E). To facilitate the tasks of mechanical pollination and yield estimation based on chili flower and bud recognition, the “8214upup” mutant chili variety was selected as the core experimental specimen. This variety exhibits a typical upright inflorescence phenotype, characterized by upward-extending floral organs and stable morphology, making it particularly suitable for constructing visual datasets oriented towards plant phenotypic analysis.

**Figure 1:**
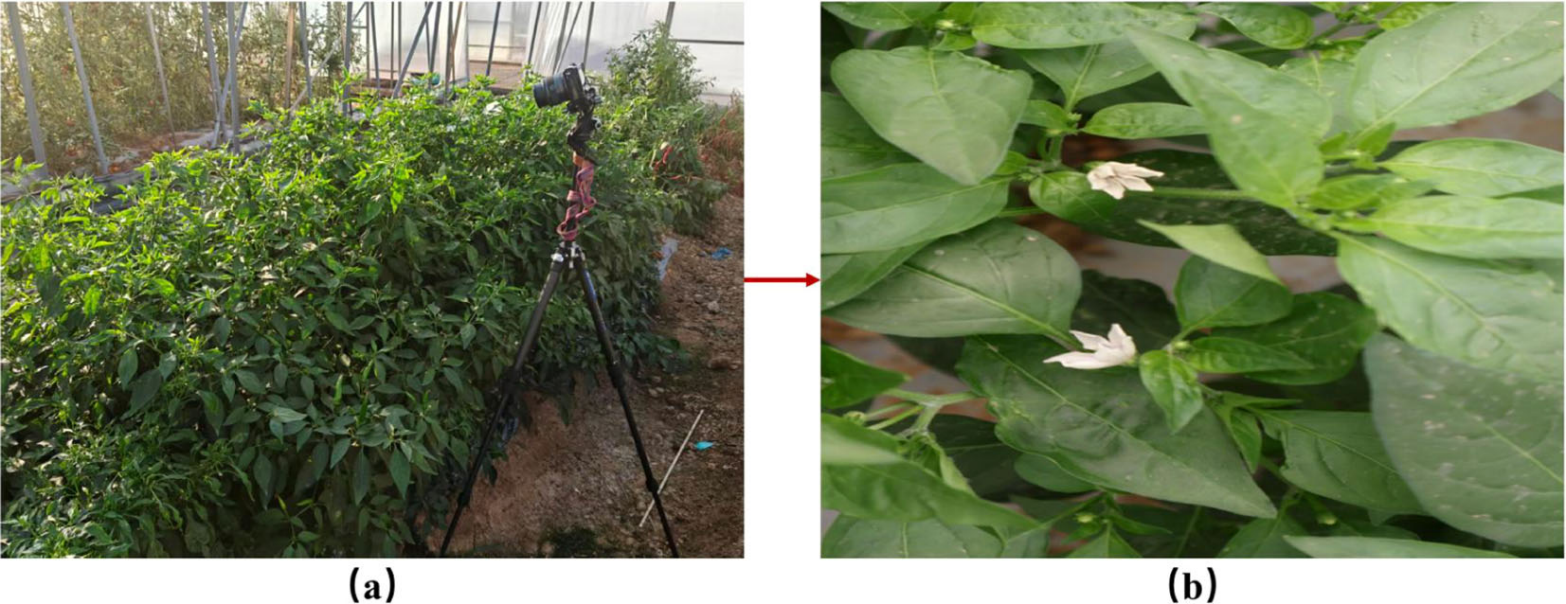
The image acquisition process and representative examples. **(A)** The left image depicts the experimental setup for field data collection using a DSLR camera with a tripod for multi-angle shooting. **(B)** The right image shows a representative sample of pepper flowers, highlighting challenges such as low background contrast and diminutive target sizes.

During the peak flowering period of chili peppers from June to August 2025, image data were collected using a Canon R50 high-definition digital camera. To ensure that the dataset adequately covered chili flower growth characteristics under diverse natural environmental conditions, image acquisition was conducted during two daily periods, namely 06:00–11:00 and 15:00–17:00, thereby capturing flower images under varying illumination conditions. During data acquisition, a multi-angle surrounding imaging strategy was employed for individual chili plants, combined with different shooting heights (approximately 30–80 cm) and viewing angles (0°–45°), to continuously record chili flowers at different developmental stages. This acquisition strategy effectively enhanced the diversity of the dataset in terms of target scale, occlusion level, and viewpoint distribution, thereby providing multi-scenario raw data support for subsequent flower recognition and growth-stage classification model development. The image acquisition equipment and representative samples are shown in **Figure 1**. Statistically, the number of chili flowers per image ranges from 1 to 9, with an average of 5 targets for each picture. The wide numerical variation manifests uneven sample distribution and complex field environments, which well reflects the practical detection difficulty of this task.

**Figure 2:**
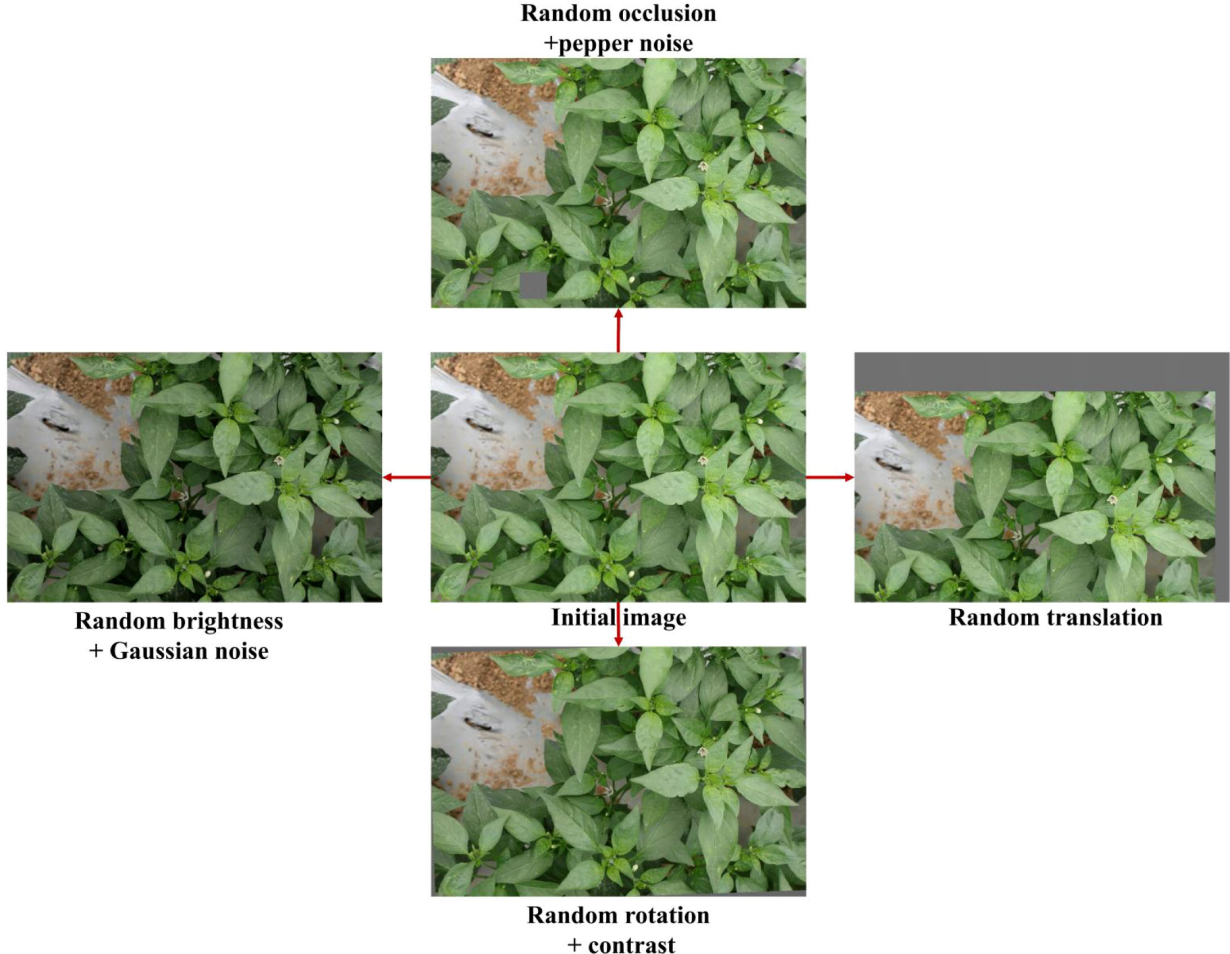
Dataset augmentation. Various augmentation techniques are applied to the “Initial image” to simulate complex field conditions: Random occlusion + pepper noise, Random translation, Random rotation + contrast adjustment, and Random brightness + Gaussian noise.

To ensure model robustness within unstructured field environments, a rigorous data cleaning and augmentation workflow was implemented. Initially, manual screening was performed to remove invalid samples suffering from severe motion blur or extreme occlusion, resulting in a baseline dataset of 1,875 high-resolution images. Subsequently, to address the limitation of single transformations in simulating complex agricultural environments, a composite data augmentation strategy was introduced (as shown in **Figure 2**). This strategy incorporated operations including random brightness adjustment with Gaussian noise, random occlusion with salt-and-chili noise, geometric translation, and random rotation with contrast adjustment. This process expanded the dataset to 5,399 images. All samples were finely annotated using the X-AnyLabeling software to generate Ground Truth labels containing spatial coordinates and categories. Finally, the dataset was partitioned into training, validation, and testing sets in an 8:1:1 ratio, comprising 4,319, 539, and 541 images, respectively.

### 2.2 Technological route of the proposed methods

**Figure 3:**
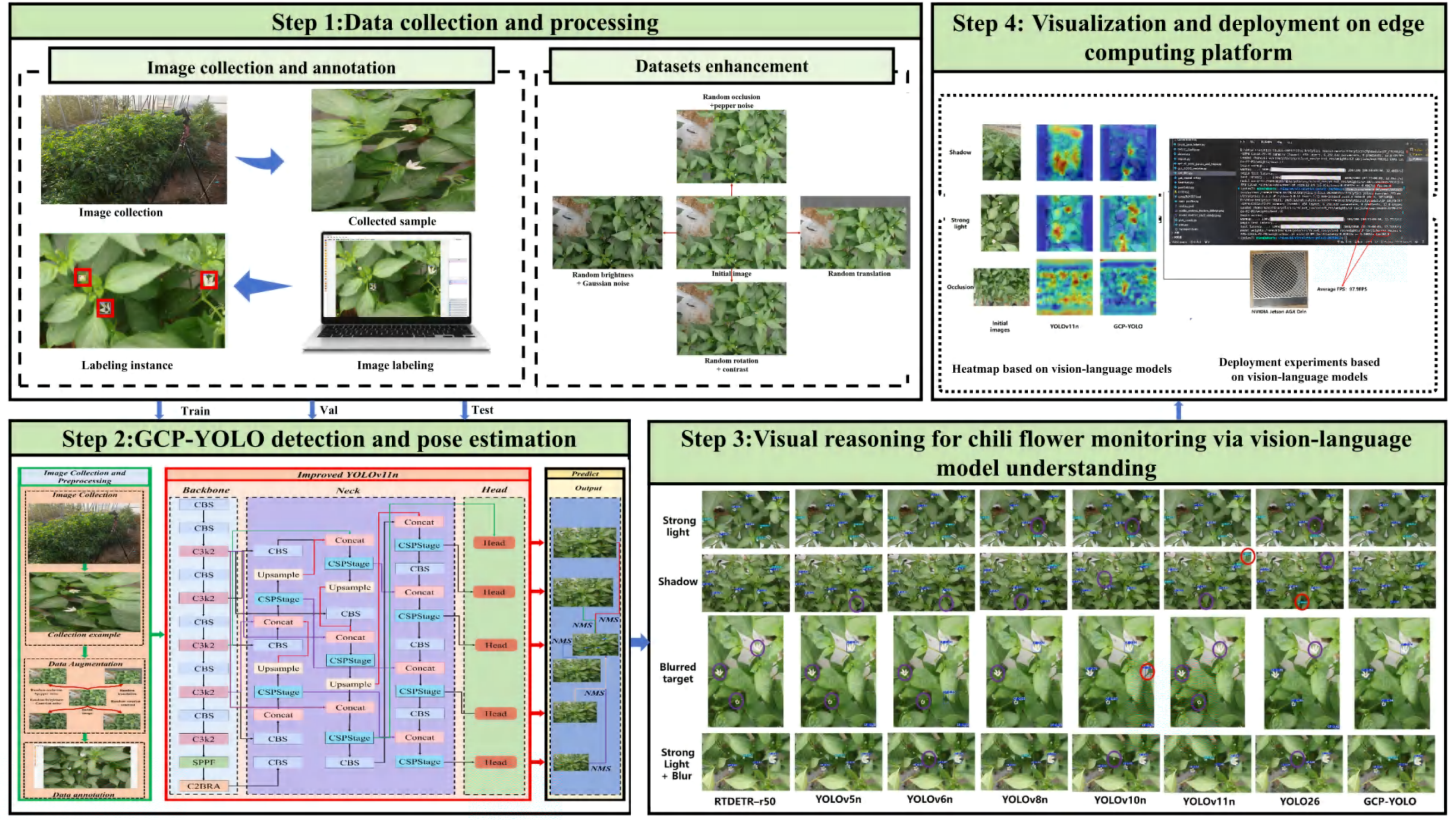
Technological route of the proposed method. The overall pipeline includes four key stages: (Step1) Data collection and processing, covering image acquisition, manual annotation, and dataset enhancement; (Step2) CFPR-YOLO detection and pose estimation, implementing the improved network architecture for chili flower detection and pose prediction; (Step3) Visual reasoning based on vision-language models, achieving multi-scenario visualization analysis and performance comparison across different detectors; (Step4) Visualization and deployment on edge computing platforms, validating the practical implementation and inference efficiency on Nvidia Jetson AGX Orin edge devices.

**Figure 3** illustrates the overall technical roadmap proposed in this study for the precise detection chili flowers and buds. The framework aims to leverage improved deep learning algorithms to overcome bottlenecks associated with limited micro-target perception and severe background interference in unstructured field environments. The research workflow systematically encompasses core stages: dataset construction and preprocessing, deep network architecture optimization, model training and validation, and multi-platform deployment.

(a) Data Collection and Processing Phase To address the challenges of variable field lighting and dense foliage occlusion in chili cultivation, multi-view and multi-temporal images were collected to construct a raw dataset. Fine-grained manual annotation was performed to label chili flower instances and their pose attributes, establishing a high-quality baseline dataset. Data augmentation techniques (e.g., random occlusion, brightness adjustment, and geometric transformation) were further applied to expand sample diversity, thereby enhancing the model’s generalization ability across complex field environments. (b) GCPYOLO Detection and Pose Estimation Phase Taking YOLOv11n as the baseline, the GCP-YOLO model was proposed for chili flower detection and pose estimation. To mitigate the feature loss of small-scale chili buds, a GFPN (Guided Feature Pyramid Network) was introduced to reconstruct the Neck network, strengthening the fusion of shallow texture features and deep semantic information via cross-scale skip connections. The C2PSA module in the original backbone was replaced by the designed C2CGA (C2f with Context-Guided Attention) module, which dynamically suppresses leaf reflections and complex background noise through context-aware attention mechanisms. Additionally, P2 and P6 multi-scale detection heads were incorporated to expand the model’s effective receptive field, alleviating false and missed detections in dense planting scenarios. (c) Visual Reasoning for Chili Flower Monitoring via Vision-Language Model Phase Systematic ablation studies and comparative experiments were conducted to validate the effectiveness of the proposed improvements. Quantitative metrics (mAP50, mAP50-95) and Grad-CAM visualization heatmaps were utilized to analyze the model’s feature focusing ability and detection precision under diverse conditions (e.g., strong light, shadow, occlusion, and blur). Cross-model comparisons with mainstream detectors (e.g., RTDETR, YOLOv5/6/8/10/11, YOLO26) further demonstrated the superiority of GCP-YOLO in balancing accuracy and efficiency for chili flower monitoring. (d) Visualization and Deployment on Edge Computing Platform Phase The trained GCP-YOLO model was deployed on an NVIDIA Jetson AGX Orin embedded edge computing platform and integrated into a local GUI visualization system. Field tests verified the model’s real-time inference performance (average 10 ms per frame) on resource-constrained devices, while heatmap visualization based on vision-language models provided intuitive interpretability for flower detection results. This implementation offers reliable technical support for intelligent chili yield estimation and robotic pollination guidance in practical agricultural scenarios.

### 2.3 Chili flower detection model

As a recent iteration of the YOLO series, YOLOv11 demonstrates excellence in general object detection tasks with strong real-time processing capabilities. However, it remains insufficient for tasks in complex, unstructured agricultural environments. Specifically, when facing scenarios where chili flower targets are minute in size, densely distributed, occluded by foliage or affected by lighting variations, the original model faces severe challenges regarding insufficient feature extraction, loss of micro-target features, and limited localization precision. To enhance the perception and monitoring capabilities for tiny flowers and buds, this study proposes a high-performance detection network named GCP-YOLO, based on the YOLOv11n architecture. Closely aligning with the characteristics of field operational environments, the model incorporates systematic innovative designs across three dimensions: feature fusion architecture reconstruction, attention mechanism optimization, and multi-scale perception expansion.

Firstly, addressing the significant scale variance of chili flowers and buds in images and the tendency of deep networks to lose shallow micro-features, the GFPN was introduced to reconstruct the Neck feature fusion structure. Unlike traditional feature pyramids, which often suffer from information attenuation during multi-scale feature aggregation, GFPN innovatively introduces cross-scale skip-connections and weighted feature fusion mechanisms. This design effectively constructs high-speed transmission channels between shallow high-resolution texture information and deep high-semantic information, reinforcing the interaction and complementarity of features at different levels. By assigning adaptive weights to features of different scales, GFPN significantly enhances the model’s perceptual acuity for the morphological details of small chili flowers and tiny buds, effectively resolving the issue of feature imbalance under the coexistence of multi-scale targets.

Secondly, to further suppress complex background noise interference, the C2CGA module was designed and introduced to replace the C2PSA module in the original backbone. Targeting the issue of blurred flower edges caused by strong leaf reflections and cluttered backgrounds in field environments, the C2CGA module utilizes a Context-Guided Attention mechanism to achieve dynamic weighting of key information during the feature extraction stage. This mechanism automatically focuses on the morphological edges and key texture features of flowers based on contextual information while significantly suppressing invalid background responses generated by uneven lighting or leaf occlusion. This improvement not only enhances the purity of feature extraction but also strengthens the model’s anti-interference ability against blurred or subtle targets while maintaining high-confidence classification.

Finally, to resolve the difficulties of false and missed detections caused by local feature confusion in dense planting environments, P2 and P6 multi-scale detection heads were added to the original detection heads. The original three-scale detection heads often lack precision when dealing with extreme scale variations. The P2 detection head, by introducing higher-resolution shallow features, focuses on capturing extremely tiny bud targets; the P6 detection head extends the model’s effective receptive field in deep layers, combining global contextual information to assist in judging target contours under complex occlusion. The collaborative operation of these five-scale detection heads fundamentally resolves discrimination difficulties brought by target overlap in dense scenarios, drastically reducing the missed detection rate caused by leaf occlusion or feature similarity, thus ensuring the integrity of the monitoring task.

**Figure 4:**
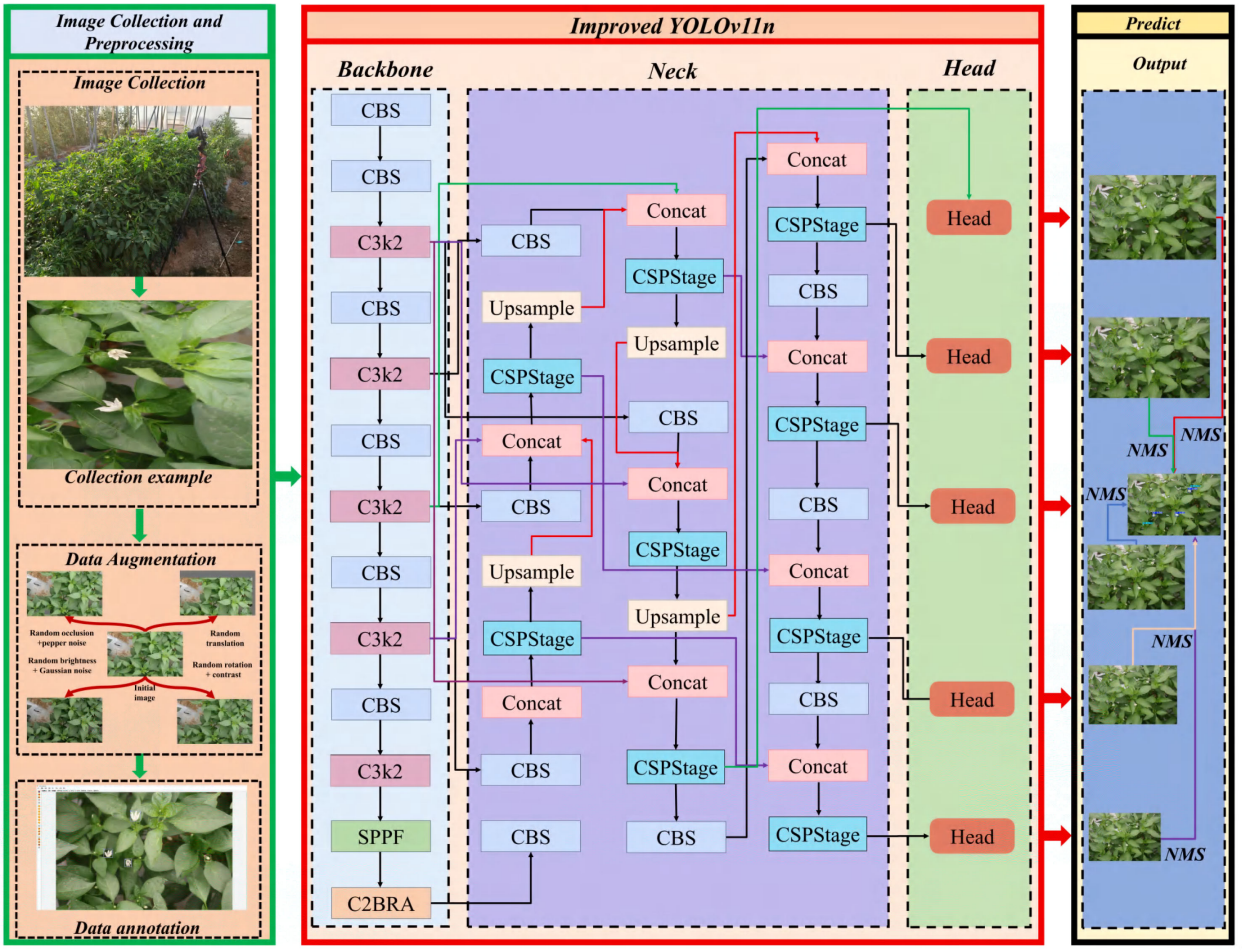
Network architecture of GCP-YOLO model. The architecture comprises a Backbone for robust feature extraction, a Neck featuring the improved GFPN for multi-scale feature fusion, and a specialized detection Head integrating P2 and P6 layers. Components like CBS, C3k2, and the C2CGA attention module are color-coded to represent their functional roles.

The structure of the improved GCP-YOLO model is shown in **Figure 4**. Through the synergistic action of these three core improvements, the model achieves high-precision and high-efficiency perception of chili flower poses in complex unstructured environments, providing solid technical support for subsequent yield estimation. Details for each of the three architectural innovations are given below.

#### 2.3.1 Generalized feature pyramid network

**Figure 5:**
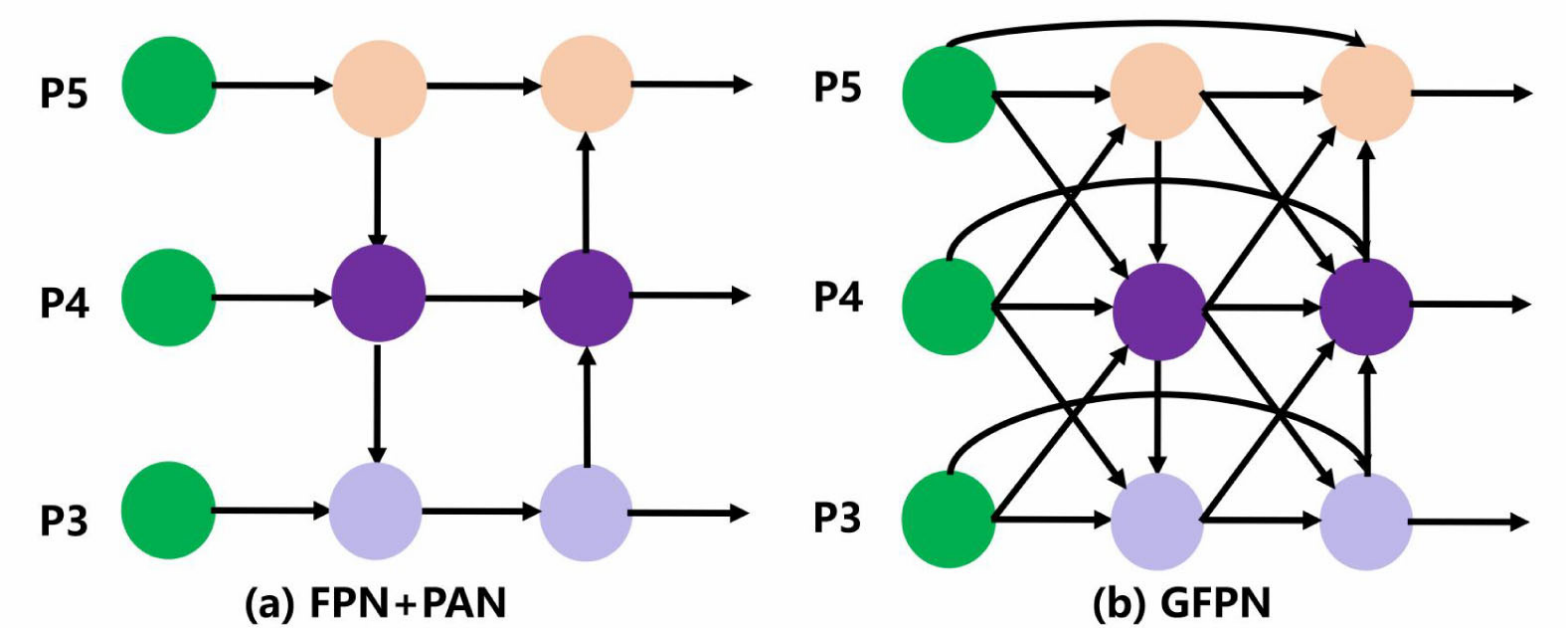
GFPN fusion module structure diagram. **(A)** The standard FPN+PAN structure with basic bidirectional information flow. **(B)** The proposed GFPN (Generalized Feature Pyramid Network) architecture, which utilizes dense skip connections to facilitate more effective cross-layer feature transmission and preserve fine-grained information of small pepper flowers.

In the original YOLOv11 architecture, although the Path Aggregation Network (PANet) adopted in the Neck introduces bidirectional path aggregation, it exhibits significant limitations in feature transmission across deep networks. The prolonged information interaction paths in traditional FPN and PANet structures cause the texture features of tiny targets, such as chili buds, to dissipate gradually through successive convolutional layers. Furthermore, reliance solely on adjacent-level fusion is inadequate for addressing the drastic scale variations encountered in unstructured field environments. To overcome this challenge, this study reconstructs the feature fusion layer by introducing a GFPN (as shown in **Figure 5**), aiming to enhance the representation capability of multi-scale features through a “Heavy-Neck” paradigm. The core innovation of GFPN lies in breaking the sparse connection constraints of traditional pyramids by constructing a full-dimensional information interaction topology via skip-layer and cross-scale connections.

Targeting the issues of gradient vanishing and feature blurring of small objects during backpropagation in deep networks, GFPN abandons the computationally redundant Dense-link pattern in favor of an innovative logarithmic connection mechanism. This mechanism establishes sparse yet efficient shortcut connections between different levels based on logarithmic laws, enabling the l-th layer feature to not only receive information from the previous layer but also directly aggregate historical features from the (*l* −2*^n^*)-th layer. The feature extraction formula is given by Equation 1:

(1) $$P_{k}^{\left(l\right)}=\text{Conv}(\text{Concat}(\{P_{k}^{\left(l-2^{n}\right)},\;P_{k}^{\left(l-1\right)}|l-2^{n}\geq 0\}))$$

where $P_{k}^{\left(l\right)}$ represents the feature map of the l-th layer at the *k*-th scale; Conv denotes the convolutional operation for dimensional integration of concatenated features; Concat refers to the feature concatenation operation along the channel dimension; n is a non-negative integer; and the constraint *l* − 2*n* ≥ 0 ensures that the index of the selected historical feature layer is a valid positive number. This logarithmic skipping mechanism injects shallow, high-resolution texture information into the deep network while ensuring computational efficiency, effectively mitigating the “semantic dissipation” phenomenon of tiny bud features in deep layers.

Moreover, to address the immense scale disparity between chili flowers and buds in images (ranging from buds of a few pixels to large flower clusters), GFPN introduces a cross-scale fusion strategy named Queenfusion. Unlike PANet, which is restricted to feature summation between adjacent scales, Queen-fusion mimics the movement rules of the “Queen” in chess, allowing the current node to simultaneously receive feature flows from the same level, the upper level (downsampling), and the lower level (upsampling). Specifically, for feature fusion at scale k, the system concurrently executes Bi-linear Upsampling to receive deep semantics from Pk+1 and Max Pooling downsampling to receive shallow details from Pk-1. This fusion mechanism not only eliminates the semantic gap between levels but also enables the network to adaptively balance the weights of features at different scales. This ensures that even under complex occluded backgrounds, the model can accurately infer the category and location of targets by leveraging robust contextual information, realizing high-precision perception of multi-pose chili flowers.

#### 2.3.2 C2CGA module

In the baseline YOLOv11n model, the C2PSA (C2f with Pyramid Squeeze Attention) module balances computational efficiency and feature interaction capabilities to some extent through a pyramid squeeze attention mechanism. However, its core relies on cross-channel partial self-attention computation. This mechanism presents limitations when processing targets with high feature specificity, such as chili flowers. Due to the diminutive size of chili flowers and their high textural similarity to leaves, excessive channel isolation weakens global context modeling capabilities. Particularly in transitions from distant to close-up views, the subtle texture features of flowers are easily overwhelmed by complex leaf backgrounds, making it difficult for the model to distinguish reflective leaves from real buds, leading to missed detections.

**Figure 6:**
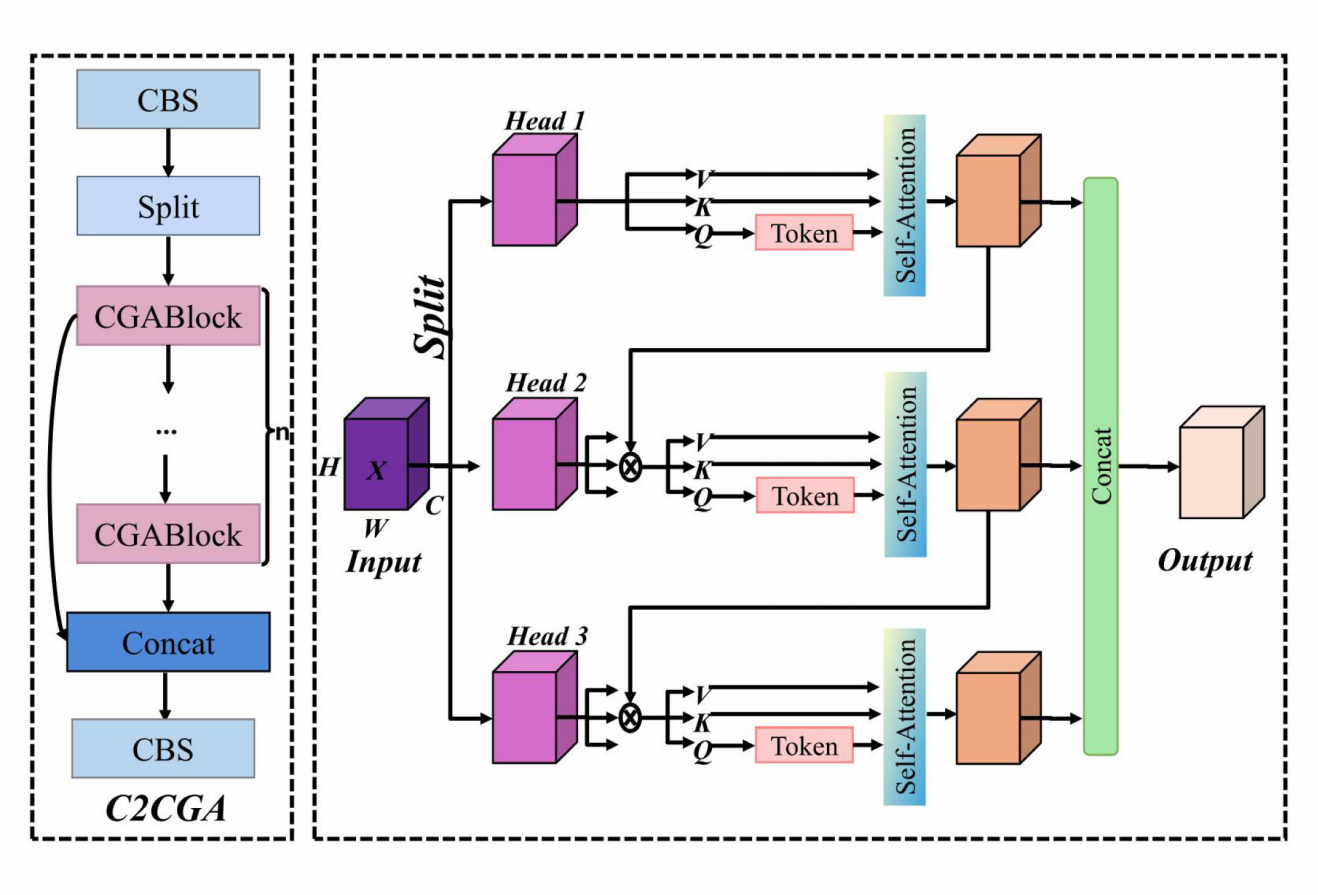
C2CGA module structure diagram. The diagram illustrates the macro-flow (Split and Concat operations) and the micro-mechanism (Multi-head Self-Attention and Token generation). This design enables the model to adaptively focus on morphological edge textures while suppressing complex agricultural background noise.

To overcome these challenges, this study introduces the C2CGA module (as shown in **Figure 6**). The core philosophy of CGA lies in the strategy of “Feature Splitting - Cascaded Interaction - Feature Reassembly,” which significantly enhances the model’s ability to capture multi-scale contextual information while reducing computational redundancy. The principle involves C2CGA splitting the input feature *X* into *h* independent feature subspaces (Heads) along the channel dimension, with each subspace computing self-attention independently. Unlike traditional Multi-Head Attention (MHA) where heads are mutually independent, CGA adopts a cascaded approach, superimposing the output features of the previous head onto the input of the next head. This design not only increases the equivalent depth of the network but also realizes the progressive refinement of features and the cumulative transmission of contextual information.

The information transmission and feature extraction process is as follows: First, for the *j*-th attention head, the input feature *X_ij_* generates query vector *Q*, key vector *K*, and value vector *V* through linear projection. The feature representation *X_ij_*of this subspace is computed using the self-attention mechanism, as shown in Equation 2:

(2) $${\tilde{X}}_{ij}=\text{Attention}(X_{ij}W_{ij}^{Q},\;X_{ij}W_{ij}^{K},\;X_{ij}W_{ij}^{V})$$

where Attention represents the self-attention computation, and $W_{ij}^{Q},W_{ij}^{K},W_{ij}^{V}$ are the projection weight matrices corresponding to the *j*-th head. Crucially, CGA introduces a cascaded interaction mechanism. Except for the first head, the input *X_ij_*’ of the *j*-th head is a weighted fusion of the currently allocated feature slice *X_ij_*and the output feature of the previous head. This progressive transmission mechanism allows subsequent attention heads to perform fine-grained corrections based on coarse-grained features extracted previously, thereby constructing complete contextual dependencies within the feature space. Its output feature expression is given by Equation 3:

(3) $${X^{\prime }}_{ij}=X_{ij}+{\tilde{X}}_{i(j-1)}$$

Finally, the output features of all h heads are concatenated and fused via a linear projection layer *W^P^* to restore the original dimensionality, thereby enabling cross-channel information integration. The cross-channel output is formulated as defined in Equation 4.

(4) $${\tilde{X}}_{out}=\text{Concat}[{\tilde{X}}_{i1},\;{\tilde{X}}_{i2},\;\ldots ,\;{\tilde{X}}_{ih}]W^{P}$$

where *X_out_*is the final output feature of the C2CGA module; Concat is the feature concatenation operation merging the output features of *h* attention heads; represents the feature output by the *j*-th attention head; and $W^{P}$ is the linear projection matrix used to restore the concatenated features to the original dimension. For the chili flower detection task, this cascaded design holds significant physical meaning: different attention heads can focus on distinct feature dimensions. For instance, earlier heads may capture the geometric contours of flowers, while subsequent heads combine contextual information (such as branch connectivity) to distinguish buds from reflective leaf noise. Through this progressive feature extraction, C2CGA effectively enhances the model’s robustness under severe occlusion and uneven lighting conditions. Furthermore, the selection of the grouping number *h* is critical to model performance. A too-small *h* value leads to high redundancy between attention heads, making it difficult to capture diverse morphological features; conversely, a too-large *h* value increases feature diversity but significantly raises computational complexity and VRAM usage, undermining the lightweight design intent. Experimental verification confirmed that setting *h* = 4 achieves an optimal balance in feature expression capability while maintaining low computational overhead. Therefore, this study adopts the configuration of *h* = 4, effectively resolving the difficulty of insufficient feature extraction for tiny buds in complex agricultural environments via the C2CGA module.

#### 2.3.3 P2 and P6 detection heads

To further improve the models ability to distinguish different target sizes, the three-scale detection heads.

(P3, P4, P5) used in the original YOLOv11n architecture still possess inherent limitations when dealing with extreme scale variations of chili flowers and buds. Addressing the minute size of field chili buds, which are prone to “feature dissipation” in deep networks, as well as the dense foliage and fruit-flower overlap in the mid-to-late growth stages, single local features are often insufficient to distinguish occluded flowers from background noise.

**Figure 7:**
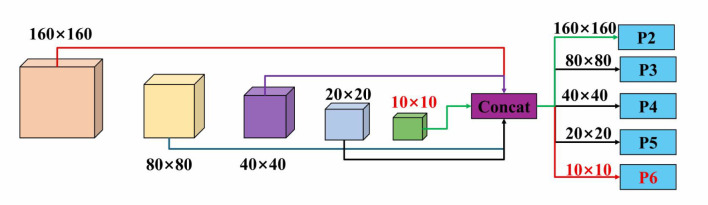
Structure of P2 and P6 head module. The diagram shows the feature map extraction process ranging from high-resolution (160×160 for P2, targeting minute buds) to large receptive fields (10×10 for P6, targeting dense clusters). Concat operations are used to integrate spatial and semantic information across different scales.

Consequently, as shown in **Figure 7**, P2 and P6 detection heads were added. The resolution of the P3 layer feature map is insufficient to support the geometric detail reconstruction of extremely tiny buds, while the P5 layer, despite possessing strong semantic information, has a receptive field that struggles to cover the complete local topological structure of the plant when facing “flower-leaf aliasing” and severe occlusion in dense greenhouse environments, limiting contextual inference capabilities. To this end, this study extended the detection architecture with P2 (4x downsampling) and P6 (64x downsampling) detection heads, constructing a full-scale perception system covering micro-textures to macro-semantics.

Assuming the input image size is H×W, the grid division granularity of the original P3 layer is H/8×W/8. When the target size O is smaller than the grid stride, multiple tiny targets may be squeezed into a single grid, leading to feature coupling. After introducing the P2 layer, the grid granularity is refined to H/4×W/4. According to the Nyquist-Shannon sampling theorem, a higher sampling rate can more accurately restore the discrete signals of tiny targets. The feature mapping relationship of the P2 detection head can be expressed as in Equation 5:

(5) $$F_{P2}=C(\text{Upsample}(F_{P3})⊕T(C_{2}))$$

where *P*3 is the feature of the *P*3 detection head; *C*_2_ is the shallow output of the backbone network; *T* is the channel transformation operation; ⊕ represents feature concatenation and fusion; *F_P_*_2_ is the feature corresponding to the *P*2 detection head; and Upsample denotes the operation increasing the resolution of *P*3 layer features. By introducing *P*2, the model can cover tiny buds with high-density anchors in shallow layers, significantly reducing the missed detection rate caused by insufficient resolution.

The P6 detection head, through further downsampling (64x), resulting in a feature map with a spatial resolution of 1/64 of the input image, drastically expands the effective receptive field of neurons, enabling the model to capture global contextual information beyond a single flower. drastically expands the effective receptive field of neurons, enabling the model to capture global contextual information beyond a single flower. This global semantic information serves as a prior constraint, assisting the model in judging the category probability of ambiguous targets. When suspected occluded flower features are detected, the contextual information provided by P6 can confirm whether the location conforms to botanical growth logic, thereby effectively suppressing false detections caused by high-light reflections on leaves. Ultimately, the constructed five-scale detection heads (P2–P6) form a multi-level pyramidal decision-making system.

### 2.4 Test environment and evaluation metrics

#### 2.4.1 Test environment

To ensure the reliability and objectivity of the experimental results, this study strictly followed the principle of variable control, establishing a standardized experimental environment. All model training, ablation studies, comparative experiments, and inference tests were completed on the same computing platform. Regarding hardware, the workstation was equipped with an Intel^®^ Core™ i5-13600KF processor and an NVIDIA GeForce RTX 3060 Ti (8GB VRAM) graphics card to provide computational support. The software architecture was based on the Windows 11 Enterprise (64-bit) operating system, configured with the CUDA 11.8 parallel computing library. Algorithm development was conducted in the PyCharm Community Edition environment, implemented using the Python 3.8 programming language and the PyTorch deep learning framework. In comparative experiments, all models maintained consistency in dataset partitioning and training strategies, employing an early stopping mechanism, with other parameters kept at their original defaults. Specific hyperparameter configurations are detailed in **Table 1**.

**Table 1:** Training hyperparameters used in the proposed model.

Parameter	Setting
Input resolution	640 × 640 × 3
Optimizer	SGD
Momentum	0.937
Early stopping patience	100
Initial learning rate	0.01
Final learning rate	0.01
Batch size	4
Number of epochs	200

∗ Other hyperparameters are set to default values.

#### 2.4.2 Model evaluation metrics

Targeting the characteristics of minute size and complex backgrounds of chili flowers and buds, and to comprehensively and objectively quantify the model’s overall performance in pose recognition and localization tasks, Precision (P), Recall (R), and mean Average Precision (mAP) were selected as core metrics for evaluating detection accuracy. Precision characterizes the accuracy of the model’s positive predictions for chili flowers, reflecting its ability to resist false detections. Recall characterizes the model’s ability to retrieve all real chili flower targets from complex backgrounds, reflecting its robustness against missed detections. mAP0.5 is the APs for all categories when the Intersection over Union (IoU) threshold is set to 0.5. mAP50–95 is a core metric in object detection tasks that comprehensively measures the model’s detection and precise localization capabilities under varying degrees of localization strictness, calculated by averaging the APs of each category at 10 IoU thresholds ranging from 0.5 to 0.95 in increments of 0.05. The mathematical definitions of these metrics are given in Equations 6–9:

(6) $$P=\frac{C_{TP}}{C_{TP}+C_{FP}}\times 100\%$$

(7) $$R=\frac{C_{TP}}{C_{TP}+C_{FN}}\times 100\%$$

(8) $$AP=\int_{0}^{1}P(R)\;dR$$

(9) $$mAP=\frac{\sum_{i=1}^{n}AP_{i}}{n}$$

where *C_TP_*(True Positives) represents the number of chili flower or bud samples correctly identified and localized by the model (i.e., IoU between predicted and ground truth boxes is greater than the set threshold); *C_FP_*(False Positives) represents the number of false detection samples, i.e., the number of predicted boxes where background noise (e.g., leaf reflection) or incorrect categories are misjudged as the current target category; and *C_FN_*(False Negatives) represents the number of missed detection samples, i.e., real chili flower or bud targets that failed to be detected by the model. AP (Average Precision) is mathematically defined as the area under the Precision-Recall (P-R) curve, used to measure the comprehensive detection performance of the model for a single category. Furthermore, to accommodate the computational constraints of the target deployment hardware, the generated model weights were configured in FP16 (half-precision) format during inference speed evaluations. This adoption of FP16 precision effectively strikes an optimal balance between detection accuracy and computational volume, accelerating real-time processing and reducing memory consumption without a perceptible loss in performance.

## 3 Results and discussion

To validate the effectiveness of the GCP-YOLO model in chili flower monitoring, this study conducted a comprehensive evaluation across four dimensions: ablation studies, comparative experiments, visual detection results, and heatmap analysis. The model’s performance was assessed in terms of detection accuracy, computational efficiency, and real-time inference capability on an NVIDIA Jetson AGX Orin embedded GPU development board, with specific metrics including frames per second (FPS), number of parameters, and FLOPs.

### 3.1 Ablation Experiment

Ablation experiments Kuang et al. (2026e) serve as a critical validation method in deep learning model optimization, assessing the contribution of individual components by systematically removing or adjusting them. This study performed a rigorous ablation analysis on the proposed GCP-YOLO model, focusing on the independent roles and synergistic effects of three core improvement modules: Module A (GFPN feature fusion structure), Module B (C2CGA module), and Module C (P2 and P6 detection head). Using YOLOv11n as the baseline, quantitative evaluations were conducted across multiple dimensions, including detection accuracy (Precision P, Recall R, mAP) and computational efficiency (Parameters, FLOPs). As shown in **Table 2**, the ablation experiments not only validated the technical effectiveness of each module but also provided a scientific basis for structural optimization.

**Table 2:** Ablation study of the proposed modules in GCP-YOLO.

Models	improved module			Efficiency			Metrics (%)			
	GFPN^*^	C2CGA^*^	P2–P6^*^	Params	FLOPs	FPS	*P*	*R*	mAP_50_	mAP_50–95_
YOLOv11n	–	–	–	2.58	6.3	104.7	90.7	82.4	86.9	65.6
YOLOv11n	✓	–	–	3.66	8.2	88.4	91.3	83.9	88.0	66.7
YOLOv11n	–	✓	–	2.56	6.3	85.3	90.5	80.8	85.6	63.6
YOLOv11n	–	–	✓	3.24	7.4	73.4	**94.1**	81.2	86.6	64.1
YOLOv11n	✓	✓	–	3.90	8.2	74.1	91.2	**85.0**	88.2	66.0
YOLOv11n	✓	–	✓	6.32	12.8	124.9	93.7	82.5	87.5	66.6
YOLOv11n	–	✓	✓	3.21	7.4	131.5	92.9	81.1	86.0	63.6
YOLOv11n	✓	✓	✓	6.29	12.8	50.3	92.8	83.7	**90.8**	**72.2**

✓ indicates that the corresponding module is enabled. *indicates the three key modules proposed in this paper. Bold text indicates the optimal result under the corresponding evaluation metric.

The asterisk symbol * denotes that the corresponding model achieves the optimal performance among all comparative algorithms under the current evaluation metric.

Analysis of **Table 2** demonstrates the individual contributions and collaborative effects of the three proposed modules. Compared with the baseline YOLOv11n, introducing GFPN alone improved Recall from 82.4% to 83.9% and increased mAP_50_ and mAP_50−95_ by 1.1 and 1.1 percentage points, respectively, indicating that the enhanced feature fusion strategy facilitates information interaction across different scales and improves the detection of small chili targets.

In contrast, incorporating C2CGA alone did not improve detection performance. Recall, mAP_50_, and mAP_50−95_ decreased to 80.8%, 85.6%, and 63.6%, respectively. This result suggests that the attention mechanism cannot fully exploit its feature enhancement capability when operating independently on the lightweight baseline architecture.

When only the P2–P6 structure was introduced, Precision increased substantially from 90.7% to 94.1%, representing the highest Precision among all configurations. However, Recall decreased slightly to 81.2%, and the mAP metrics remained close to the baseline. These results indicate that extending the feature pyramid improves localization confidence for small objects but does not sufficiently enhance overall detection completeness when used alone.

The combination of GFPN and C2CGA achieved the highest Recall of 85.0%, outperforming the baseline by 2.6 percentage points. This demonstrates that the two modules provide complementary advantages in feature aggregation and contextual representation. However, the corresponding mAP_50_ and mAP_50−95_ improvements remained limited, suggesting that further optimization of multi-scale localization capability was still required.

The GFPN+P2–P6 configuration increased Precision to 93.7% while maintaining competitive Recall and mAP performance. In contrast, the C2CGA+P2–P6 combination failed to produce comparable gains, further confirming that C2CGA relies on enhanced multi-scale feature representation to achieve its full effectiveness.

When all three modules were integrated, the proposed GCP-YOLO achieved the best overall performance with an mAP_50_ of 90.8% and an mAP_50−95_ of 72.2%, improving by 3.9 and 6.6 percentage points over the baseline, respectively. Although the parameter count increased from 2.58 M to 6.29 M and the computational complexity rose from 6.3 GFLOPs to 12.8 GFLOPs, the model maintained a real-time inference speed of 50.3 FPS. These results demonstrate that the three modules exhibit strong complementarity and jointly improve feature extraction, target localization, and multi-scale representation capabilities, resulting in the optimal balance between detection accuracy and deployment efficiency.

### 3.2 Comparative Experiments

#### 3.2.1 Comparison of different backbone network alternatives

To systematically investigate the impact of different feature fusion structures on model performance for chili flower detection and yield estimation, eight sets of comparative experiments were designed. The original PANet feature fusion structure in YOLOv11n was replaced by BiFPN, HSFPN, EMBSFPN, GDFPN, BiMAFPN, CGRSFPN, and GFPN, respectively. The experimental results are presented in **Table 3**.

**Table 3:** Performance comparison of different neck architectures for chili flower detection (all values in %).

Neck architecture	P	R	mAP50	mAP50-95
PANet	90.7	82.4	86.9	65.6
BiFPN	89.2	82.1	86.0	64.8
HSFPN	92.7	80.2	86.0	64.0
EMBSFPN	92.0	80.0	87.0	65.7
GDFPN	**94.4**	80.2	87.1	66.4
BiMAFPN	94.0	79.6	86.9	65.3
CGRSFPN	91.7	81.6	86.3	64.4
GFPN (ours)∗	**91.3**	**83.9**	**88.0**	**66.7**

∗ It indicates the proposed neck architecture in this work. Bold text indicates the optimal result under the corresponding evaluation metric.

The asterisk symbol * denotes that the corresponding model achieves the optimal performance among all comparative algorithms under the current evaluation metric.

Analysis of **Table 3** reveals significant performance differences among feature fusion mechanisms when processing tiny targets. While GDFPN and BiMAFPN structures excelled in Precision, reaching 94.4% and 94.0% respectively, their Recall dropped drastically to 80.2% and 79.6%, significantly lower than the baseline’s 82.4%. This imbalance of “high precision, low recall” indicates an overly conservative detection strategy that suppresses candidate targets with indistinct features, resulting in substantial missed detections of occluded or shadowed buds. This severely deviates from the core requirement of detection integrity in yield estimation tasks. Furthermore, BiFPN and CGRSFPN failed to surpass the baseline model in comprehensive detection accuracy, suggesting limitations in their feature aggregation strategies for tiny targets in unstructured field backgrounds.

In contrast, the model incorporating the GFPN structure demonstrated comprehensive performance. GFPN not only maintained a high Precision of 91.3% but also achieved the highest Recall among all models, improving by 1.5 percentage points over the baseline. Its Recall (R), mAP50, and mAP50–95 reached 93.9%, 88.0%, and 66.7%, respectively, ranking best across the board. These results suggest that GFPN, through its unique cross-scale skip connections and dense connection mechanisms, effectively strengthens the interaction between deep semantic information and shallow high-resolution texture features, significantly mitigating feature loss of tiny buds during downsampling. By ensuring high localization precision while minimizing the missed detection rate, GFPN achieves the best balance between detection integrity and accuracy, and was thus selected as the final feature fusion structure for this study.

#### 3.2.2 Comparative experiments incorporating replacements for C2PSA

To explore the influence of replacing different C2PSA modules on model performance, five sets of comparative experiments were designed. Based on the GFPN-integrated model, the original C2PSA module in the backbone was replaced by CASSD, C2BRA, C2DA, and C2CGA modules, respectively. The results are shown in **Table 4**.

**Table 4:** Comparison of attention modules replacing C2PSA in the backbone (all values in %).

Backbone variant	P	R	mAP50	mAP50:95
YOLOv11n + A	91.3	83.9	88.0	**66.7**
YOLOv11n + CASSA	90.7	83.7	88.0	65.9
YOLOv11n + CBRA	90.8	83.4	87.3	65.9
YOLOv11n + C2DA	**92.8**	81.6	87.5	66.1
YOLOv11n + C2CGA (ours)∗	91.2	**85.0**	**88.2**	66.0

∗ It indicates the proposed attention module in this work. Bold text indicates the optimal result under the corresponding evaluation metric.

The asterisk symbol * denotes that the corresponding model achieves the optimal performance among all comparative algorithms under the current evaluation metric.

The data indicate that although the C2DA module achieved the highest Precision of 92.8%, its Recall plummeted to 81.6%. This significant P-R imbalance suggests excessive suppression of candidate targets, leading to the omission of numerous blurred or tiny buds, which is detrimental to detection integrity. Meanwhile, CASSD and C2BRA modules failed to outperform the baseline model (YOLOv11n+A) in key metrics, indicating ineffective enhancement of anti-interference capabilities against complex backgrounds.

Conversely, the model incorporating the C2CGA module exhibited the best overall detection performance. While its Precision remained comparable to the baseline, Recall significantly increased to 85.0%, a 1.1 percentage point improvement, and mAP50 reached a peak of 88.2%. This demonstrates that the ContextGuided Attention mechanism in C2CGA effectively captures morphological edges and key texture features of flowers and buds. It maintains high-confidence classification while drastically reducing missed detections caused by leaf occlusion or reflections. Although mAP50–95 showed slight fluctuations, considering that target discovery (high Recall) is more critical than extreme bounding box regression precision in agricultural monitoring applications, the C2CGA module aligns better with practical requirements. Therefore, C2CGA was selected as the core improvement for the backbone network, working in conjunction with the GFPN structure to build a high-precision detection model.

#### 3.2.3 Comparison of different detection head configurations

To systematically investigate the impact of different detection head configurations, four sets of comparative experiments were conducted based on the model integrated with GFPN and C2CGA. Additional detection heads P2, P6, and the P2+P6 combination were added, respectively. The results are presented in **Table 5**.

**Table 5:** Impact of multi-scale detection head extensions on model performance (all values in %).

Model variant	P	R	mAP50	mAP50:95
YOLOv11n + GFPN + C2CGA	91.2	**85.0**	88.2	66.0
+ P2 head	**95.4**	81.1	87.5	65.9
+ P6 head	95.1	80.3	87.1	66.0
+ P2 + P6 heads (ours)∗	92.8	83.7	**90.8**	**72.2**

∗ It indicates the multi-scale detection head extension proposed in this work. Bold text indicates the optimal result under the corresponding evaluation metric.

The asterisk symbol * denotes that the corresponding model achieves the optimal performance among all comparative algorithms under the current evaluation metric.

The data analysis shows that introducing the P2 or P6 detection head individually yielded significant advantages in Precision, rising to 95.4% and 95.1%, respectively. However, Recall dropped substantially to 81.1% and 80.3%, respectively, compared to the baseline. This pronounced P-R imbalance indicates that while a single-scale detection head enhances discrimination for specific target scales, it leads to excessive suppression of chili buds with non-salient features or large-scale differences, causing massive missed detections. Furthermore, neither configuration showed significant improvement over the baseline in mAP metrics, suggesting that single-level feature enhancement is insufficient to cope with variable field interference.

In contrast, the model utilizing the P2+P6 combination detection heads demonstrated improved detection performance. This configuration not only maintained a high Precision of 92.8% but also achieved substantial improvements in average precision, with mAP50 rising to 90.8% and mAP50–95 reaching 72.2%. The experimental results indicate that the synergistic action of P2 and P6 detection heads effectively fuses detailed features of tiny buds with morphological features of large-scale flowers, greatly enhancing detection accuracy across different IoU thresholds. Given that high-precision target localization is crucial for subsequent fruit monitoring and segmentation in yield estimation tasks, the performance gains from the P2+P6 combination meet practical application needs. Consequently, this study determined to simultaneously add P2 and P6 modules to the detection head section.

#### 3.2.4 Comparison with other models

To evaluate the overall performance of the proposed GCP-YOLO model in chili flower pose recognition, comparative experiments were conducted against current mainstream object detection algorithms, including RTDETR-r50, YOLOv5n, YOLOv6n, YOLOv10n, YOLOv11n, YOLO26n, and GCP-YOLO.

We selected these mainstream models as baselines because they represent the state-of-the-art in lightweight detection and offer strong generalization capability across diverse domains, including agriculture. While specialized models exist for plant or flower detection, they often require domain-specific training data or architectural adaptations, which would limit comparability under our unified evaluation protocol. All models were trained and tested using the same dataset and hardware platform with consistent parameter configurations to ensure objectivity and fairness. The results are shown in **Table 6**.

**Table 6:** Comparison with representative detection models for greenhouse chili monitoring (all values in %).

Detector	P	R	mAP50	mAP50-95	Params (M)	FLOPs (G)	FPS(f/s)
RTDETR-R50	91.8	82.3	86.6	61.2	42.00	136.0	18.5
YOLOv5n	93.4	81.9	87.2	66.6	1.93	4.5	135.2
YOLOv6n	**93.7**	82.9	87.2	67.2	4.37	11.1	118.4
YOLOv10n	93.3	80.2	86.1	67.8	3.23	8.7	126.3
YOLOv11n	90.7	82.4	86.9	65.6	2.58	6.3	104.7
YOLO26n	90.0	78.5	83.3	60.6	2.38	5.2	89.1
GCP-YOLO (Ours)∗	92.8	**83.7**	**90.8**	**72.2**	62.9	12.8	50.4

∗ It indicates the proposed improved GCP-YOLO model in this work. Bold text indicates the optimal result under the corresponding evaluation metric. Params, number of parameters; FLOPs, floating point operations; FPS, frames per second.

The asterisk symbol * denotes that the corresponding model achieves the optimal performance among all comparative algorithms under the current evaluation metric.

The comparison reveals substantial differences in both detection accuracy and computational efficiency among the evaluated models. YOLOv5n, YOLOv6n, and YOLOv10n achieved relatively high Precision values of 93.4%, 93.7%, and 93.3%, respectively. However, their Recall values remained below 83%, and their mAP_50−95_ scores did not exceed 68%, indicating limited capability in capturing challenging chili flower targets under complex greenhouse conditions. RTDETR-R50 achieved competitive Precision and Recall, but its mAP_50−95_ reached only 61.2%. In addition, the model required 42.00 M parameters and 136.0 GFLOPs, resulting in the lowest inference speed of 18.5 FPS among all compared methods. YOLOv11n and YOLO26n exhibited relatively low Recall values of 82.4% and 78.5%, respectively, accompanied by inferior mAP performance.

From the perspective of deployment efficiency, YOLOv5n, YOLOv6n, YOLOv10n, YOLOv11n, and YOLO26n maintained high inference speeds ranging from 89.1 to 135.2 FPS owing to their lightweight architectures. However, the reduction in model complexity was accompanied by a decline in localization accuracy, particularly for the mAP_50−95_ metric. RTDETR-R50 achieved stronger feature representation capability but introduced substantially higher computational cost, which may limit its applicability on resource-constrained agricultural robotic platforms.

Compared with the competing models, the proposed GCP-YOLO achieved the best overall detection performance, obtaining a Recall of 83.7%, an mAP_50_ of 90.8%, and an mAP_50−95_ of 72.2%. Although its Precision of 92.8% was slightly lower than that of YOLOv6n, the model delivered superior performance in Recall and mAP metrics.

Specifically, GCP-YOLO improved mAP_50_ by 4.2 percentage points compared with RTDETR-R50, by 3.6 percentage points compared with YOLOv6n, and by 3.4 percentage points compared with YOLOv11n. Moreover, its mAP_50−95_ exceeded that of the strongest baseline model, YOLOv10n, by 4.4 percentage points and outperformed RTDETR-R50 by 11.0 percentage points. These improvements indicate that the proposed architecture effectively enhances feature extraction and localization accuracy for small and densely distributed chili flowers under complex greenhouse conditions.

Although GCP-YOLO increased the computational cost to 12.8 GFLOPs, it still maintained a real-time inference speed of 50.4 FPS, which is substantially higher than the practical requirement for robotic harvesting applications. Compared with RTDETR-R50, the proposed model reduced computational complexity by approximately 90.6% while simultaneously improving mAP_50−95_ by 11.0 percentage points. These results demonstrate that GCP-YOLO achieves a favorable balance between detection accuracy and deployment efficiency.

To rigorously assess the model’s resilience, robustness testing was performed using four stochastically distributed seeds (Default, 1, 42, and 3407). Statistical analysis revealed that the standard deviations for P, R, mAP50, and mAP50–95 were minimal (0.27, 0.06, 0, and 0.49, respectively), demonstrating that GCP-YOLO is highly insensitive to stochastic initialization. In the context of smart farming, this level of stability is critical; it guarantees that the perception system delivers invariant and dependable phenotypic data across heterogeneous hardware deployments and varying operational windows, eliminating the risk of decision-making errors triggered by algorithmic instability. To further evaluate the stability and robustness of the proposed model, experiments were conducted under multiple random initialization seeds, and the corresponding results are summarized in **Table 7**.

**Table 7:** Robustness evaluation of GCP-YOLO under different random initialization seeds (all values in %).

Seed	Performance				Standard deviation (std)			
	*P*	*R*	mAP_50_	mAP_50:95_	*P*	*R*	mAP_50_	mAP_50:95_
Default	92.8	83.7	90.8	72.2				
1	93.3	83.8	90.8	71.5				
42	93.3	83.8	90.8	71.5	0.27	0.06	0.00	0.49
3407	93.3	83.3	90.8	71.5				

The remarkable reproducibility of the model across random seeds provides empirical rigor for large-scale yield estimation. With coefficients of variation (CV) as low as 0.27% for Precision and 0.06% for Recall, the system demonstrates superior reliability in generating consistent output. In particular, the zero-variance performance in mAP50 underscores the model’s deterministic reliability in core detection tasks, offering a potent guarantee for yield forecasting, resource optimization, and economic impact assessments in chili cultivation.

### 3.3 Visualization of detection results

**Figure 8:**
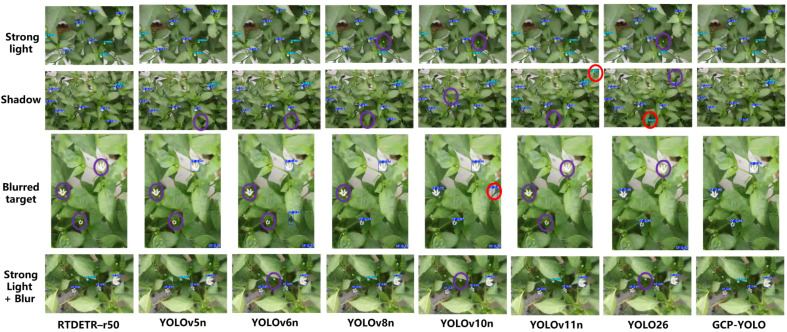
Comparison of the visual detection effect of each model under different lighting conditions. Evaluation under Strong light, Shadow, Blurred target, and composite environments. Purple circles indicate successful precise detections by GCP-YOLO, while red circles highlight common failure points (missed or false detections) in traditional models like RT-DETR and YOLOv5n through 26n.

**Figure 9:**
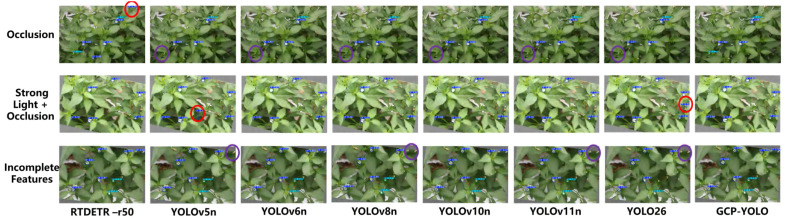
Comparison of the visual detection effect of each model under different occlusion conditions. Comparative analysis under Occlusion, Strong light + Occlusion, and Incomplete features. The red circles mark failed cases where competing models lacked sufficient feature reconstruction capability, whereas GCP-YOLO demonstrates superior robustness in morphological completion.

The performance of object detection models relies heavily on the quality of datasets. In indoor agriculture environments, the photographic conditions are complex, often affected by uneven lighting and occlusion, leading to a limited quantity and unbalanced distribution of original image samples, which in turn impacts model training efficacy. To enhance model robustness and generalization capability, this study conducted a comparative analysis of the visual detection results of the GCP-YOLO model on a test set partitioned after applying various augmentation strategies. The comparative visual detection results under different lighting and occlusion conditions are shown in **Figures 8**, **9**, respectively.

Firstly, from the perspective of lighting variations and imaging quality, different models exhibited significant disparities in adaptability to unstructured environments. As illustrated, in test scenarios such as “strong light,” “shadow,” and “blur,” YOLOv5n, YOLOv6n, YOLOv8, YOLOv11, YOLO26, and RTDETR-r50 showed high missed detection rates (indicated by purple circles). This is primarily due to the loss of target surface texture features caused by intense lighting variations or low contrast in shadowed areas, making it difficult for traditional feature extraction networks to effectively separate targets from the background. Furthermore, in the “blurred target” scenario, YOLOv10n even exhibited obvious false detections (red circles), misidentifying background noise as targets. In contrast, the improved GCP-YOLO model, leveraging its enhanced feature extraction capability, maintained extremely high detection acuity even in such low signal-to-noise ratio environments, effectively circumventing missed and false detections caused by uneven lighting and motion blur.

Secondly, complex background interference, particularly foliage occlusion, is a critical factor constraining the accuracy of field object detection. In the “occlusion” and “strong light + occlusion” test groups, mainstream algorithms (including YOLOv8n, YOLOv11n, and YOLO26) appeared inadequate when handling overlapping targets, frequently missing detections (purple circles). Meanwhile, RTDETR-r50 and YOLO26 failed to accurately distinguish leaf edges from real targets when processing complex background textures, resulting in false positive misdetections (red circles). Conversely, the GCP-YOLO model, benefiting from its optimized feature fusion mechanism, effectively suppressed background feature interference. Even under complex conditions where targets were largely occluded by foliage or situated in high-highlight backgrounds, it accurately captured the semantic features of targets, demonstrating excellent anti-occlusion performance.

Finally, in extreme edge scenarios such as “incomplete features” and “edge targets,” the robustness comparison among models was even more intuitive. For targets located at image edges or having incomplete features due to shooting angles, the YOLOv5n to YOLOv11n series models universally suffered from insufficient feature aggregation capabilities, leading to bounding box regression failures and severe missed detections. However, the GCP-YOLO model, through the introduction of improvement modules, strengthened the perception of local incomplete features and the integration of contextual information, successfully achieving precise localization of edge-incomplete targets. In summary, the visualization results powerfully prove that GCP-YOLO possesses significantly superior generalization capabilities and detection precision compared to existing mainstream models under variable environments, complex occlusions, and extreme imaging conditions, making it more suitable for actual agricultural production environments.

### 3.4 Heat map detection image comparison

**Figure 10:**
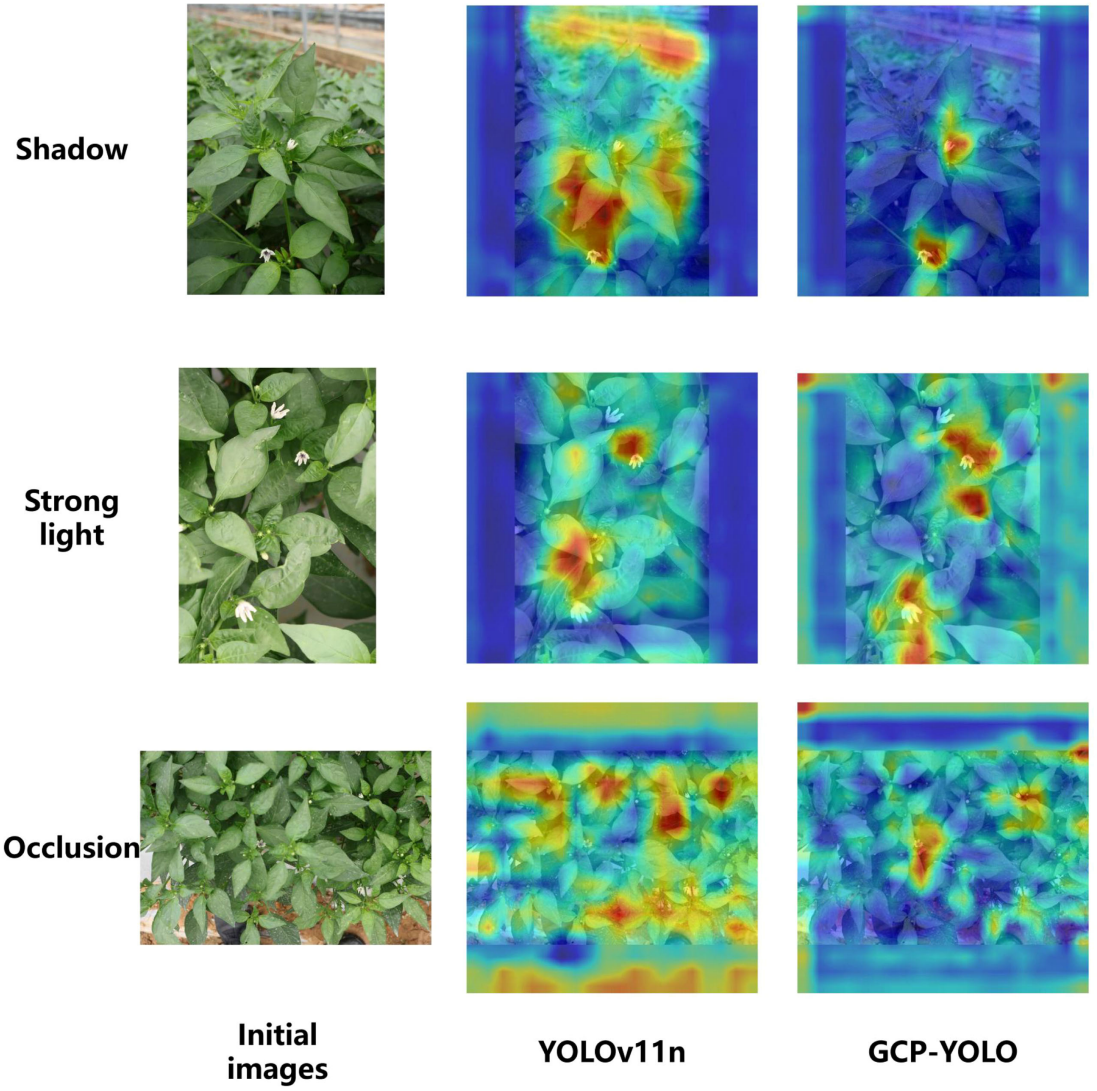
Comparison of heatmap visual detection effect of each model under different scenes. Comparison of activation maps between the baseline YOLOv11n and the proposed GCP-YOLO. The results indicate that GCP-YOLO (right column) focuses more accurately on the target centers in Shadow and Occlusion scenes, effectively mitigating interference from surrounding foliage.

To explore the feature extraction logic of the improved algorithm in complex unstructured environments from the perspective of model interpretability, this study used Grad-CAM (Gradient-weighted Class Activation Mapping) technology to generate class activation heatmaps. By comparing the thermal response distributions of the baseline YOLOv11n and the improved GCP-YOLO model under typical field scenarios (shadow, strong light, dense occlusion) as shown in **Figure 10**, significant differences in feature attention points were revealed.

From the visualization results, the original YOLOv11n model (middle column) exhibited a clear phenomenon of “attention diffusion”. In shadow and strong light scenarios, its high-response regions (dark red areas) failed to precisely cover the main body of the flowers and instead overflowed substantially onto surrounding leaf edges and branch backgrounds. This indicates that the baseline model is highly susceptible to interference from environmental light changes and waxy leaf reflections, making it difficult to isolate effective flower texture features from complex backgrounds. particularly in dense occlusion scenarios (third row), the thermal distribution of YOLOv11n presented a disordered, fragmented state, showing a severe attenuation of its feature extraction capability under heavy occlusion. Essentially, the single-path feature pyramid of original YOLOv11n lacks sufficient cross-layer information interaction, resulting in mismatched shallow contour and deep semantic features and uncontrollable scattered attention regions.

In contrast, the GCP-YOLO model (right column) demonstrated exceptional capabilities in “target focusing” and “background suppression.” Whether in low-contrast shadow regions or high-reflection strong light environments, the thermal peaks of the improved model were highly concentrated on the petal edges and pistil centers of the chili flowers, with clear and convergent activation boundaries that matched the geometric contours of real targets. Even under severe occlusion conditions with interlaced foliage, the model precisely locked onto tiny bud targets, effectively suppressing weight responses in non-target regions. Mechanically, the customized GFPN realizes bidirectional multi-scale feature aggregation to compensate the missing fine-grained flower feature of single-scale feature extraction, while embedded C2CGA coordinate attention adaptively suppresses redundant weight assignment on irrelevant leaf and branch background pixels from spatial dimension. This highly differentiated visual performance demonstrates that by introducing GFPN and C2CGA attention mechanisms, the model successfully achieved efficient integration of deep semantics and shallow textures, significantly enhancing the discriminative power for tiny key features, thereby fundamentally improving detection robustness in complex agricultural environments.

### 3.5 Deployment experiments

To assess the real-world deployment viability of the proposed model on resource-constrained agricultural robots, inference tests were conducted on an NVIDIA Jetson AGX Orin embedded edge platform. The model, equipped with GFPN, C2CGA, and multi-scale detection heads, demonstrates remarkable inference efficiency using FP16 precision. Throughout repeated testing, the frame rate consistently remained within a narrow margin of 96.9 to 98.9 FPS, achieving an overall average speed of 97.9 FPS. This performance corresponds to a single-frame processing latency of roughly 10 ms, easily exceeding the conventional 30 FPS benchmark necessary for real-time visual servoing. Additionally, the optimized architecture remains exceptionally compact, with only 6.29 M parameters and 12.8 GFLOPs. Ultimately, these results substantiate that the model successfully balances a significant boost in detection accuracy with superior inference efficiency and compactness, providing the low-latency, high-fluency visual feedback crucial for high-speed robotic tasks in complex, dynamic agricultural settings.

### 3.6 Error analysis

**Figure 11:**
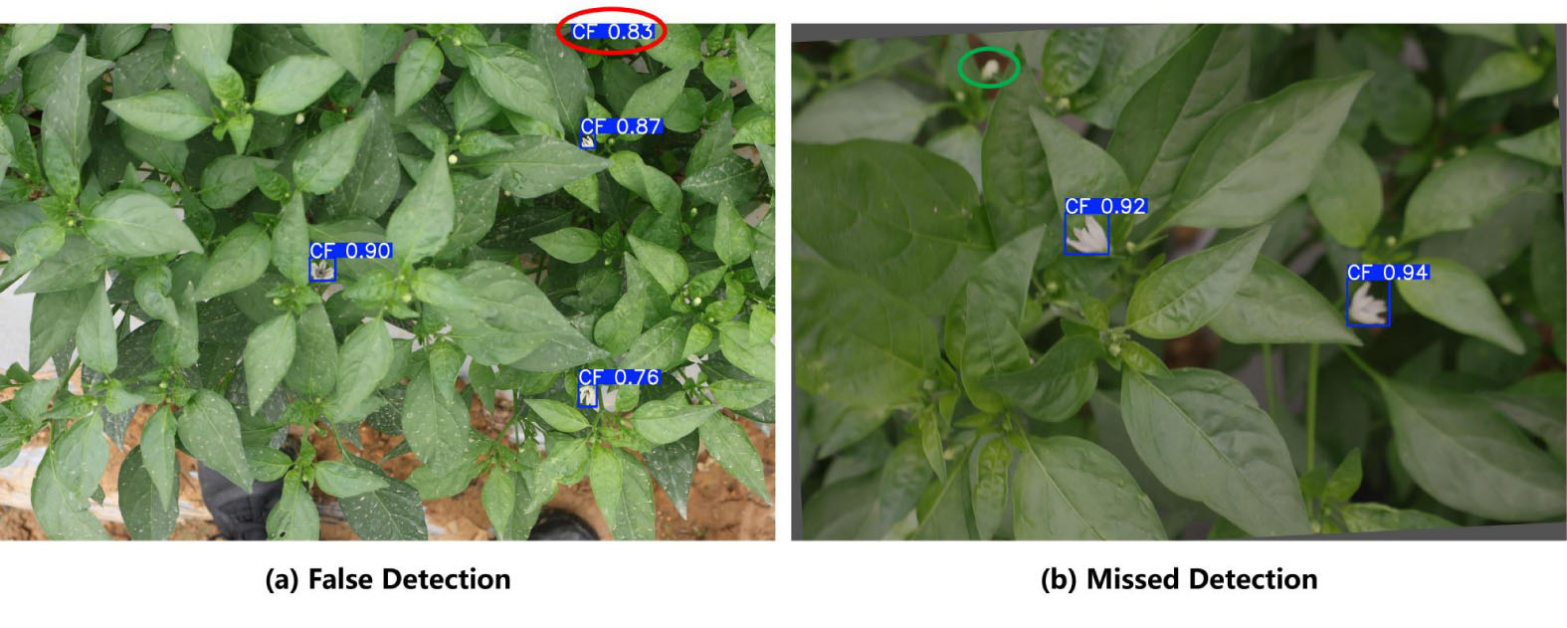
Analysis of false detection and missed detection cases. **(A)** False detection: Red circles indicate background elements (e.g., reflections or leaf textures) misidentified as flowers due to high morphological similarity. **(B)** Missed detection: Green circles show extremely small or heavily overlapped targets that the model failed to recognize. These cases provide insights for further iterative optimization.

Although the proposed GCP-YOLO achieves strong overall performance, several typical failure cases are observed under challenging environmental conditions. **Figure 11** illustrates representative examples of false detections and missed detections.

False positives mainly occur in regions with strong leaf reflections or along the edges of senescent leaves. In these cases, certain background textures exhibit morphological patterns similar to floral structures, which may confuse the detector. Missed detections are primarily associated with extremely small buds located in deep shadow or heavily occluded by surrounding foliage. Under such conditions, the visual signal is weak and feature contrast becomes insufficient for reliable recognition.

These observations indicate that extremely small targets and severe illumination variations remain challenging factors for RGB-based detection systems in greenhouse environments. Future improvements may benefit from incorporating additional sensing information or exploiting temporal cues from image sequences to further enhance detection robustness.

## 4 Conclusion and future work

To address the challenges of dense distribution, severe occlusion, and insufficient feature extraction for small chili flowers in complex greenhouse environments, this study proposed GCP-YOLO, an improved detection framework based on YOLOv11n. By redesigning the feature fusion structure, introducing a context-guided attention mechanism, and extending multi-scale detection layers, the proposed model enhanced the perception capability for small and densely distributed chili flowers under complex lighting and background conditions. The main conclusions are summarized as follows:

1. A high-precision chili flower detection framework was developed for complex agricultural environments. Experimental results showed that the precision, recall, mAP50, and mAP50–95 of the proposed GCP-YOLO model reached 92.8%, 83.7%, 90.8%, and 72.7%, respectively. Compared with the original YOLOv11n model, the corresponding metrics improved by 2.1, 1.3, 3.9, and 6.6 percentage points, demonstrating the effectiveness of the proposed improvements in enhancing small-target feature representation and dense-object perception.
2. The redesigned GFPN structure and the introduced C2CGA attention mechanism effectively strengthened cross-scale feature interaction and contextual information extraction. The model achieved more accurate localization and recognition of chili flowers under conditions involving illumination variation, background interference, and partial occlusion, thereby improving detection stability in complex greenhouse scenarios.
3. Deployment experiments on the NVIDIA Jetson AGX Orin platform demonstrated that the proposed model maintained stable real-time inference performance on edge devices, achieving an inference speed of 97.9 FPS. The results indicate that the proposed framework has good practical applicability for intelligent agricultural monitoring and robotic perception tasks.
4. Future research will further investigate the integration of multi-modal sensing information, such as depth and infrared data, to improve perception robustness under extreme illumination and heavy occlusion conditions. In addition, combining keypoint localization with robotic motion planning will be explored to achieve accurate pollination-point positioning and support the development of autonomous pollination systems in smart agriculture.

## Data Availability

The raw data supporting the conclusions of this article will be made available by the authors, without undue reservation.
